# The risk of cardiovascular disease following breast cancer by Framingham risk score

**DOI:** 10.1007/s10549-018-4723-0

**Published:** 2018-02-28

**Authors:** Sofie A. M. Gernaat, Jolanda M. A. Boer, Desiree H. J. van den Bongard, Angela H. E. M. Maas, Carmen C. van der Pol, Rhodé M. Bijlsma, Diederick E. Grobbee, Helena M. Verkooijen, Petra H. Peeters

**Affiliations:** 1 Department of Epidemiology, Julius Center for Health Sciences and Primary Care, University Medical Center Utrecht, Utrecht University, Utrecht, The Netherlands; 2 Centre for Nutrition, Prevention and Health Services, National Institute for Public Health and the Environment (RIVM), Utrecht University, Bilthoven, Utrecht, The Netherlands; 3 Department of Radiation Oncology, University Medical Center Utrecht, Utrecht University, Utrecht, The Netherlands; 4 Department of Cardiology, Radboud University Medical Center Nijmegen, Nijmegen, The Netherlands; 5 Department of Surgical Oncology, Utrecht University, Utrecht, The Netherlands; 6 Department of Medical Oncology, University Medical Center Utrecht, Utrecht University, Utrecht, The Netherlands; 7 Imaging Division, University Medical Center Utrecht, Utrecht University, Utrecht, The Netherlands; 8 Department of Epidemiology and Biostatistics, School of Public Health, Imperial College, London, UK; 9 Present Address: Utrecht, The Netherlands

**Keywords:** Breast cancer, Cardiovascular disease, Mortality, Morbidity, Risk

## Abstract

**Objectives:**

This study evaluates the risk of cardiovascular disease (CVD) following breast cancer, accounting for baseline CVD risk.

**Methods:**

Within the EPIC-NL (Dutch part of the European Prospective Investigation into Nutrition and Cancer) cohort, 1103 women were diagnosed with breast cancer. For every breast cancer patient, 3–4 women without breast cancer (*n* = 4328) were selected matched for age, year, and time since cohort enrollment. Based on CVD risk factors at cohort enrollment, 10-year risk of CVD was calculated and categorized: low (< 10%), intermediate (10–20%), high (> 20%). Cox proportional hazard models assessed the risk of CVD events (hospitalization or mortality) and CVD mortality of women with versus without breast cancer, adjusted for baseline CVD risk.

**Results:**

After median follow-up of 5 and 6 years, 92 (8.3%) and 325 (7.5%) CVD events occurred in women with and without breast cancer, respectively. In the low CVD risk group, women with breast cancer had 1.44 (95% CI 1.00–2.06) times higher risk of CVD events than women without breast cancer. In the intermediate and high CVD risk categories, risk of CVD events was similar in women with and without breast cancer. Overall, women with breast cancer had 1.77 (95% CI 1.10–2.86) times higher risk of CVD mortality than women without breast cancer.

**Conclusions:**

Among women with low CVD risk, women with breast cancer have a higher risk of CVD event than women without breast cancer. Overall, women with breast cancer have a higher risk of CVD mortality than women without breast cancer.

**Electronic supplementary material:**

The online version of this article (10.1007/s10549-018-4723-0) contains supplementary material, which is available to authorized users.

## Introduction

Breast cancer incidence and survival are high in developed countries [1]. Survival has substantially improved due to early detection by screening programs and improved treatments [2–4]. This has resulted in over 3 million 5-year breast cancer survivors worldwide [1]. Many of these women will die of conditions other than breast cancer [5, 6]. Cardiovascular disease (CVD) is an important cause of death in the general population, also following breast cancer with 24% of patients over 65 years die of this disease [7, 8].

Breast cancer patients may have a higher CVD risk compared to the general population. Although cancer treatments such as anthracycline-based regimens, trastuzumab, and radiotherapy reduce the risk of cancer recurrence and death, they have been associated with an increased risk of CVD [9–16]. Anthracycline-based chemotherapy and trastuzumab increase the risk of heart failure by fivefold compared to regimens without these components [17, 18]. Furthermore, radiotherapy increases the risk of death from circulatory disease with 25% [11]. Another reason that breast cancer patients may have a higher CVD risk is because risk factors for both diseases overlap, especially risk factors as obesity and physical inactivity [19]. Breast cancer patients may have a higher prevalence of CVD risk factors than the general population. Pre-existing CVD risk factors have also been associated with a higher risk of cancer treatment-induced cardiotoxicity [20, 21].

Except for a few studies [7, 22, 23], the majority did not adjust for traditional CVD risk factors when investigating the risk of CVD following breast cancer. The Framingham risk score is a composite score based on traditional CVD risk factors (age, sex, current smoking, diabetes, and high systolic blood pressure) to predict the absolute 10-year baseline CVD risk [24]. The current study assessed the risk of CVD for women with breast cancer, compared to women without breast cancer, with a low (< 10%), intermediate (10–20%), and high (> 20%) baseline risk of CVD. Next, we assessed the risk of death from CVD adjusted for the baseline CVD risk.

## Methods and materials

### Study design and population

The current study included women participating in the Dutch contribution to the European Prospective Investigation into Cancer and Nutrition (EPIC-NL), which consists of the MORGEN and Prospect cohorts [25]. Details on the design and rationale of the EPIC-NL study have been described elsewhere [26]. Briefly, prospect is a prospective cohort study that was set up to investigate the role of nutrition and biomarkers in the etiology of cancer. The MORGEN cohort was set up to monitor risk factors for chronic diseases in the Netherlands. MORGEN includes 22,654 men (45%) and women aged 20–64 years residing in three Dutch towns (Amsterdam, Doetinchem, and Maastricht) between 1993 and 1997 [27]. Prospect includes 17,357 women aged 49–70 living in the city of Utrecht or its vicinity who participated in the nationwide Dutch breast cancer screening program between 1993 and 1997 [28]. The ethics committees of the respective institutions approved both studies, and all participants gave their written informed consent.

Women with prevalent cancer at EPIC-NL enrolment (*t*0) were not eligible for the current study. Furthermore, women were not included if they had not given consent for linkage with vital status or morbidity registries (*n* = 2717) or had missing information on hospital admission or cause of death (*n* = 55). The current study included all women diagnosed with a first in situ or invasive breast cancer during follow-up in the EPIC-NL cohort until December 31, 2010 (referred to as ‘exposed’ in the current study). Subsequently four women without breast cancer during follow-up were matched to the exposed women on age at breast cancer diagnosis (*t*1), year of breast cancer diagnosis (*t*1), and time between EPIC-NL enrolment (*t*0) and breast cancer diagnosis (*t*1) (the ‘unexposed’ group). We would like to stress that this is not a matched case–control study, but rather a prospective follow-up study, matched on the exposure status (breast cancer).

The final study population consisted of 1103 women diagnosed with breast cancer and 4328 women without breast cancer.

### Exposure (breast cancer) assessment

In situ or invasive breast cancers in the EPIC-NL study were identified through regular linkages to the Dutch Cancer Registry. Details on the registry linkage have been described elsewhere [26]. Briefly, the Dutch Cancer Registry identifies incident cancer cases by hospital records and is 95% complete since 1989.

### Characteristics

At *t*0, a general questionnaire was filled out by all participants including questions on demographic characteristics, presence of chronic diseases, and risk factors for chronic diseases. Educational level was categorized into low (primary school and lower vocational education) and other (advanced elementary education, intermediate vocational education, higher general secondary education, higher vocational education, and university). Diabetes was present if participants were diagnosed with diabetes according to the general questionnaire. Physical activity was assessed by questions on occupational and recreational physical activity during the past year at *t*0 [29]. The Cambridge Physical Activity Index combined these activities and categorized them into active, moderately active, moderately inactive, and inactive [30, 31]. Smoking behavior was categorized into current, former, or never. Alcohol consumption (gram ethanol per day) was assessed with a validated Food Frequency Questionnaire at *t*0 [32, 33]. The body mass index was calculated as weight divided by height squared (kg/m^2^), which were measured during physical examination. At this contact, blood pressure was measured twice in supine position on the left arm using a random zero sphygmomanometer (MORGEN) and on the right arm using a Boso Oscillomat (Prospect), from which the mean was taken. The comparability of both measurement procedures is reported in more detail elsewhere [34]. In MORGEN, serum cholesterol levels were assessed from ethylene–diamine–tetra–acetic acid (EDTA) serum samples drawn during physical examination at *t*0 using an enzymatic method [26]. In Prospect, cholesterol values are measured with EDTA using serum samples and/or with citrate plasma.

History of CVD before *t*1 was determined by combining data from the general questionnaire at *t*0 and data from the Dutch Centre for Health Care Information on hospital discharge diagnosis. The Dutch Centre for Health Care Information holds a standardized computerized register of hospital discharge diagnoses coded according to the International Classification of Diseases, Ninth Revision, Clinical Modification (ICD-9).

### Framingham risk score

The Framingham risk score was calculated for the total study population based on the following characteristics at *t*0 (median of 8 years before breast cancer diagnosis or reference date): age, smoking behavior (current or past/never), diabetes (presence or absence), systolic blood pressure (mmHg), total cholesterol (mmol/L), and high-density lipoprotein (HDL) cholesterol (mmol/L) [24]. The Framingham risk score ranges from −2 to 21, indicating a 10-year absolute risk of developing CVD and risk of individual CVD events (hospitalization or death) of less than 1% to over 30%, respectively. The current study categorized the Framingham risk score into three categories: low (score: < 13, risk: < 10%), intermediate (score: 13–17, risk: 10–20%), high (score: > 17, risk: > 20%).

### Outcome assessment

The outcomes were a CVD event, defined as a hospitalization for CVD or death from CVD, and death from CVD. Follow-up data on the outcomes were complete until December 31, 2010. Follow-up data on CVD hospitalizations were obtained from the Dutch Centre for Health Care Information. The database was linked to the cohort on the basis of birth date, gender, postal code, and general practitioner with a validated probabilistic method [35]. Causes of death were obtained from the Statistics Netherlands and have been coded according to the Ninth Revision of the International Classification of Diseases (ICD-9) until 1996, and after that, according to the Tenth Revision of the International Statistical Classification of Diseases (ICD-10). Death from CVD was based on primary and secondary causes of death. The primary cause of death is defined as the underlying disease that led to death. The secondary cause of death is either a complication of the primary cause, or another disease which might have contributed to the death.

### Data analyses

Multiple imputation of missing values was performed using 20 imputed datasets to deal with missing values of demographics and cardiovascular risk factors at *t*0 [36]. In the current study, determinants with missing values were educational level (*n* = 16, 0.3%), smoking behavior (*n* = 4, 0.1%), diabetes (*n* = 6, 0.1%), systolic blood pressure (*n* = 21, 0.4%), total cholesterol (*n* = 289, 5.3%), HDL cholesterol (*n* = 296, 5.5%), alcohol consumption (*n* = 16, 0.3%), and body mass index (*n* = 6, 0.1%).

Means [standard deviation (SD)] and medians [interquartile range (IQR)] were used to describe continuous variables with and without normally distributed data, respectively. Time at risk started at *t*1 and ended at the date of a CVD event (primary outcome) or date of death from CVD (secondary outcome), death from any other cause, end of study (December 31, 2010), or loss to follow-up (*n* = 29), whichever occurred first. Cox proportional hazard models [37] were used to estimate (adjusted) hazard ratios (HR) and 95% confidence intervals, comparing women with breast cancer to women without breast cancer. In addition, a competing risk analysis [38] was performed to deal with breast cancer as a competing risk: here the HR estimated by the Fine-Gray model account for the fact that women who died of breast cancer are no longer eligible of experiencing the event of interest.

The analyses on the risk of a CVD event (hospitalization or death due to CVD) were performed for the total study population and stratified by low (< 10%), intermediate (10–20%), or high (> 20%) Framingham risk category. The analysis including the total study population was adjusted for Framingham risk score and body mass index. The analysis stratified by Framingham risk category was adjusted for age at *t*1, i.e., the stratification by Framingham risk created new groups and therefore women within these groups were no longer age-matched, and body mass. The analysis on the risk of death from CVD was performed only for the total study population and adjusted for Framingham risk score; the low number of deaths did not allow for stratification by Framingham risk category. In addition, a sensitivity analysis was performed excluding women with a history of CVD to test the hypothesis that women with a known risk of CVD at breast cancer diagnosis receive less cardiac toxic breast cancer treatments.

Statistical analyses were conducted using IBM SPSS statistics version 23, except for the competing risk analyses which were conducted with SAS version 9.4.

## Results

At EPIC-NL cohort enrolment (*t*0), median age of the study population was 54 years (IQR = 50–60) for women with breast cancer and women without breast cancer (Table 1). At *t*0, median Framingham risk score was not different for women who would develop breast cancer (13, IQR = 9–16) than for women who would not develop breast cancer (12, IQR = 9–16) (Table 1). The majority of women with and without breast cancer were in the low Framingham risk category: 61.3 and 66.0%, respectively. The mean body mass index at *t*0 was comparable for women with and without breast cancer in the low Framingham risk category: 25.2 (SD = 3.7) and 25.1 (SD = 3.8), respectively (Supplemental material Table A). In the intermediate and high Framingham risk categories, the mean body index was also comparable between women with breast cancer (27.3 (SD = 4.2) and 28.5 (SD = 3.8), respectively) and without breast cancer (27.2 (SD = 4.2) and 28.1 (SD = 4.5), respectively).

**Table 1:** Characteristics of 1103 women with breast cancer and 4328 matched women without breast cancer at time of original cohort (EPIC-NL) enrolment (*t*0) and at time of breast cancer diagnosis or reference (*t*1)

	Women with breast cancer	Women without breast cancer
	*n* = 1103	*n* = 4328
At time of original cohort enrolment (*t*0)		
Original cohort, % (*n*)		
Prospect	70.4 (776)	68.9 (2984)
MORGEN	29.6 (327)	31.1 (1344)
Age at *t*0, year, median (IQR)	54 (50–60)	54 (50–60)
Low education, % (*n*)^a^	45.2 (499)	43.9 (1898)
Physical activity, % (*n*)		
Inactive	8.2 (90)	6.2 (270)
Moderately inactive	26.5 (292)	25.0 (1080)
Moderately active	25.7 (284)	27.1 (1174)
Active	39.6 (437)	41.7 (1804)
Smoking behavior, % (*n*)		
Current	25.5 (281)	24.7 (1069)
Former	36.2 (400)	32.6 (1413)
Never	38.3 (422)	42.7 (1846)
Alcohol consumption, g/day, mean (SD)	10.3 (13.7)	9.1 (12.4)
Diabetes, % (*n*)	2.4 (27)	2.0 (86)
Systolic blood pressure, mmHg, mean (SD)	130.8 (20.3)	128.6 (20.0)
Total cholesterol, mmol/L, mean (SD)	5.9 (1.1)	5.9 (1.1)
HDL cholesterol, mmol/L, mean (SD)	1.5 (0.4)	1.5 (0.4)
Body mass index, kg/m^2^, mean (SD)	25.7 (4.1)	25.2 (4.1)
Framingham risk score, median (IQR)^b^	13 (9–16)	12 (9–16)
Framingham risk categories, % (*n*)^b^		
< 10%	61.3 (676)	66.0 (2856)
10–20%	29.1 (321)	26.1 (1131)
20%	9.6 (106)	7.9 (341)
At time of breast cancer diagnosis or reference (*t*1)		
Age at t1, year, median (IQR)	63 (56–68)	63 (56–68)
Calendar year of t1, A2		
1993–1999	26.7 (294)	26.6 (1153)
2000–2005	38.3 (422)	38.3 (1658)
2006–2010	35.1 (387)	35.1 (1517)
History of cardiovascular disease at t1, % (*n*)	68 (6.2)	219 (5.1)
Time between *t*0 and t1, year, median (IQR)	8 (4–11)	8 (4–11)
Follow-up time since t1 (until end of study), year, median (IQR)	5 (2–9)	6 (3–10)

Women were matched by (1) age at original cohort enrolment (*t*0) and (2) time between original cohort enrolment and breast cancer diagnosis (*t*1–*t*0)

*IQR* interquartile range, *SD* standard deviation

^a^Low educational level: lower vocational training or primary school

^b^Framingham risk score is based on age at original cohort enrolment, smoking behavior, diabetes, systolic blood pressure, and total and HDL cholesterol

Breast cancer patients had 5 years (IQR = 2–9) median follow-up (since *t*1) and this was 6 years (IQR = 3–10) for women without breast cancer (Table 1). During this period, 72 women with breast cancer (6.5%) and 290 without breast cancer (6.7%) were hospitalized for CVD (Table 2). Hospitalizations for acute pulmonary heart disease and heart failure were more common in women with breast cancer than in women without breast cancer. There were 24 women with breast cancer (2.2%) and 57 women without breast cancer (1.3%) who died of CVD as primary or secondary cause. Coronary heart disease and cerebrovascular accident were the most common causes of death from CVD in both groups. Death from breast cancer was the most prevalent cause of death among women with breast cancer (*n* = 115, 10.4%).

**Table 2:** Cardiovascular disease hospitalization and/or death and other causes of death in 1103 women with breast cancer and 4328 matched women until December 31, 2010

	ICD-9	ICD-10	Women with breast cancer [% (*n*)]	Women without breast cancer [% (*n*)]
Hospitalization for CVD			6.5 (72)	6.7 (290)
Coronary heart disease	410–414, 427.5, 798.1, 798.2, 798.9	I20–I25, I46, R96	2.5 (28)	3.2 (137)
Cerebrovascular accident	430–434, 436	I60–I67, I69, G45	1.1 (12)	2.1 (89)
Acute pulmonary heart disease	415	I27	1.3 (14)	0.3 (12)
Heart failure	428	I50	0.8 (9)	0.5 (22)
Arterial embolism and thrombosis	444	I74	0.3 (3)	0.3 (11)
Other	440–443	I70–I73	0.5 (6)	0.4 (19)
Death from CVD^a^			2.2 (24)	1.3 (57)
Coronary heart disease	410–414, 427.5, 798.1, 798.2, 798.9	I20–I25, I46, R96	0.4 (4)	0.5 (23)
Cerebrovascular accident	430–438	I60–I67, I69, G45	0.7 (8)	0.4 (16)
Other	401, 415, 417, 424.1, 424.2, 424.9, 425, 427.3, 427.9, 428, 440, 441, 456	I10, I26, I27, I35, I36, I38, I48, I49.9, I50, I70, I71, I85	1.1 (12)	0.4 (18)
Primary cause of death other than CVD			14.6 (161)	3.0 (170)
Breast cancer	174	C50	10.4 (115)	0.0 (0)
Other type of cancer	140–173, 175–232, 234–239	C00–C49, C51–C97, D00–D49	2.6 (29)	2.3 (98)
Other	All other codes	All other codes	1.5 (17)	1.6 (72)

Numbers may overlap as women with CVD morbidity may have died of CVD (*n* = 4 or *n* = 22 for women with or without breast cancer, respectively) or due to another cause

Women were matched by (1) age at original cohort enrolment (*t*0) and (2) time between original cohort enrolment and breast cancer diagnosis (*t*1–*t*0)

*CVD* cardiovascular disease, *ICD* international classification of diseases

^a^Primary and/or secondary CVD causes of death. 9 women with breast cancer and 8 women without breast cancer died of CVD as secondary cause of death

The risk of a CVD event (hospitalization or death due to CVD) did not differ between women with breast cancer and women without breast cancer: adjusted HR = 1.16 (95% CI 0.92–1.47) (Table 3). However, in the low Framingham risk category the risk of a CVD event was higher in women with breast cancer than in women without breast cancer: adjusted HR = 1.44 (95% CI 1.00–2.06). Excluding women with a history of CVD slightly increased this risk (Supplemental material Table B). Furthermore, in the total study population, the risk of death from CVD was higher in women with breast cancer than in women without breast cancer: adjusted HR = 1.77 (95% CI 1.10–2.86). The competing risk analyses did not change the interpretation of the results described above (Supplemental material Table C).

**Table 3:** The risk of cardiovascular disease hospitalization and/or death following breast cancer for the total study population and by low, intermediate or high Framingham risk prior to diagnosis until at most December 31, 2010

	Number of women	Total PY	Number of CVD (%)^a^	Number of CVD per 100 PY	Unadjusted HR^b^	Adjusted HR^b,c^	Adjusted HR^b,d^
CVD event (hospitalization or death)							
Total study population (*n* = 5431)							
Women without breast cancer	4328	28,035	325 (7.5)	1.2	1	1	1
Women with breast cancer	1103	6401	92 (8.3)	1.4	1.23 (0.97–1.55)	1.17 (0.92–1.57)	1.16 (0.92–1.47)
Framingham risk < 10% (*n* = 3532)							
Women without breast cancer	2856	18,518	129 (4.5)	0.7	1	1	1
Women with breast cancer	676	3783	39 (5.8)	1	1.45 (1.01–2.07)	1.45 (1.01–2.08)	1.44 (1.00–2.06)
Framingham risk 10–20% (*n* = 1452)							
Women without breast cancer	1131	7187	124 (10.9)	1.7	1	1	1
Women with breast cancer	321	2031	30 (9.3)	1.5	0.86 (0.57–1.29)	0.88 (0.59–1.32)	0.88 (0.59–1.32)
Framingham risk > 20% (*n* = 447)							
Women without breast cancer	341	2287	68 (19.9)	3	1	1	1
Women with breast cancer	106	584	23 (21.7)	3.9	1.27 (0.78–2.07)	1.27 (0.78–2.07)	1.27 (0.78–2.06)
Death from CVD							
Total study population (*n* = 5431)							
Women without breast cancer	4328	29,207	57 (1.3)	0.2	1	1	–
Women with breast cancer	1103	6717	24 (2.2)	0.4	1.88 (1.16–3.03)	1.77 (1.10–2.86)	

*CVD* cardiovascular disease, *HR* hazard ratio, *PY* person-years

^a^Row percentages of number of women

^b^Cox proportional hazard models

^c^Models including the total study population are adjusted for Framingham risk score and models stratified by Framingham risk category are adjusted for age at breast cancer diagnosis or reference (age at *t*1)

^d^Models including the total study population are adjusted for Framingham risk score and body mass index. Models stratified by Framingham risk category are adjusted for age at breast cancer diagnosis or reference (age at *t*1) and body mass index

## Discussion

The results of this study indicate that the risk of a CVD event (hospitalization or death) among women with a low Framingham risk (< 10%) is 44% higher in women with breast cancer compared to women without breast cancer. No difference was observed in the total study population. We did find that women with breast cancer have an adjusted 77% higher risk of death from CVD than women without breast cancer.

Although breast cancer is the main cause of death in women with breast cancer, CVD is increasingly recognized as an important contributor to mortality in breast cancer survivors [39–41]. CVD may be related to cardiac toxic or metabolic effects of some breast cancer treatments such as trastuzumab, anthracycline-based regimens, and radiotherapy [9, 42–44].

Several CVD disorders may contribute to a higher CVD risk following breast cancer. Women with breast cancer in the low Framingham risk category were more often hospitalized with heart failure or acute pulmonary heart disease than low-risk women without breast cancer. Heart failure is a known complication induced by anthracycline-based chemotherapies, trastuzumab, and radiotherapy-induced cardiomyopathy due to coronary artery calcifications caused by high radiotherapy heart dose [45–47]. Acute pulmonary heart disease can be caused by vascular changes as a result of tissue damage due to radiotherapy, as part of the lungs is irradiated [48]. Both heart failure and radiation-induced pulmonary damage may become evident during the first year after treatment or later [48, 49]. We also observed that women with breast cancer died more often due to a cerebrovascular accident. Women who received hormonal treatment (tamoxifen) had a 90% higher risk of a cerebrovascular accident [50]. Studies reported conflicting results on the association between cerebrovascular accident and radiotherapy to the supraclavicular lymph nodes: Nilsson et al. [51] found a 12% higher risk for women with a history of breast cancer, while Hooning et al. [21] did not found a higher risk in women with breast cancer [50, 51].

A study that stratified women by CVD risk at breast cancer diagnosis showed that in the low CVD risk group, women treated with radiotherapy were not at increased risk of CVD [52]. However, CVD risk was increased for women with an intermediate or high CVD risk [52]. These results are, however, difficult to compare with ours as a comparison with women without breast cancer is lacking. Our finding of a higher risk of CVD death in women with breast cancer is in line with many other studies [7, 39, 53, 54]. Riihimaki et al. showed that women with breast cancer have a 1.29 time higher risk of dying of heart failure [53]. They did, however, not correct for CVD risk factors other than age. Bradshaw et al. reported a 1.9 times increased risk of CVD death in women with breast cancer, after adjustment for traditional CVD risk factors [7]. This risk manifested approximately 7 years after diagnosis. Furthermore, studies have found increased risk of CVD events up to and beyond 20 years after diagnosis [8, 9, 55]. Age is a well-known CVD risk factor [56] and cardiac toxicity induced by radiotherapy manifest itself many years following treatment [15, 57]. As the current study has a relative short follow-up time (median of 5–6 years), this may indicate that the risk of death from CVD in breast cancer patients may become larger over time.

There is also a suggestive clarification for the observations in our study. The sensitivity analysis shows that the risk of a CVD event in women with breast cancer with a low Framingham risk score was higher when women with a history of CVD were excluded. This may indicate that in clinical practice women with a higher CVD risk, i.e., history of CVD, receive less cardiac toxic cancer treatments than women without a higher CVD risk [58]. As such, women with breast cancer in the low Framingham risk category may have received more often systemic therapy, i.e., anthracyclines and trastuzumab, and radiotherapy (including differences in laterality of the irradiated breast and targeted volumes) than women with an intermediate or high Framingham risk. Unfortunately, we were not able to test other hypotheses related to breast cancer treatment and characteristics as this information is missing for over one-third of patients.

We were not able to account for changes in CVD risk factors after EPIC enrolment. We assume that these factors used for calculating the Framingham risk score remained more or less similar until time of breast cancer diagnosis (*t*1) and thereafter. However, CVD risk factors may have changed between *t*0 and *t*1 (median time of 8 years) and after *t*0. This would result in women shifting to another Framingham risk category. It Is unclear how this would affect our results. Another concern is that we likely have missed women with CVD. The use of hospital discharge registry underestimates the true incidence rates, especially for coronary heart disease and heart failure [59]. This underestimation is most likely nondifferential and therefore not creating bias, as it can be expected that the underestimated incidences are not different for women with breast cancer than for women without breast cancer.

To conclude, this study shows that among women with a low Framingham risk, women with breast cancer have a higher risk of a CVD event (hospitalization or death) than women without breast cancer. Overall, women with breast cancer have a higher risk of death from CVD than women without breast cancer adjusted for Framingham risk score. Future research may investigate an individualized approach for breast cancer patients to optimize the balance between high breast cancer tumor control and minimal cancer treatment-induced CVD risk.

## Electronic supplementary material

Below is the link to the electronic supplementary material.

Supplementary material 1 (DOCX 31 kb)
