# Supplementary material for: The risk of cardiovascular disease following breast cancer by Framingham risk score
Source: Breast Cancer Res Treat. 2018 Feb 28;170(1):119–27. doi: 10.1007/s10549-018-4723-0 (PMC5993849; doi:10.1007/s10549-018-4723-0)
Supplement: Supplementary file 1 — Supplementary material 1 (DOCX 31 kb) [file 10549_2018_4723_MOESM1_ESM.docx]

## Supplemental material

### Table A. Characteristics of 1,103 women with breast cancer and 4,328 matched* women without breast cancer at time of original cohort (EPIC-NL) enrolment (t0) and at time of breast cancer diagnosis or reference (t1) by low, intermediate, and high Framingham risk

|  | **Framingham risk < 10%** | | **Framingham risk 10% - 20%** | | **Framingham risk > 20%** | |
| --- | --- | --- | --- | --- | --- | --- |
|  | **Women with breast cancer** | **Women without breast cancer** | **Women with breast cancer** | **Women without breast cancer** | **Women with breast cancer** | **Women without breast cancer** |
|  | **n = 676** | **n = 2,856** | **n = 321** | **n = 1,131** | **n = 106** | **n = 341** |
| **The following data were collected at time of original cohort enrolment (t0)** | | | | | | |
| **Original cohort, % (n)** |  |  |  |  |  |  |
| Prospect | 59.6 (403) | 59.8 (1,707) | 84.7 (272) | 85.6 (968) | 95.3 (101) | 90.6 (309) |
| MORGEN | 40.4 (273) | 40.2 (1,149) | 15.3 (49) | 14.4 (163) | 4.7 (5) | 9.4 (32) |
| **Age at t0, yr, median (IQR)** | 52 (49-57) | 52 (49-57) | 58 (54-64) | 59 (54-64) | 63 (58-67) | 63 (58-67) |
| **Low education, % (n) †** | 39.2 (265) | 38.1 (1,088) | 57.0 (183) | 53.9 (610) | 49.1 (52) | 59.2 (202) |
| **Physical activity, % (n)** |  |  |  |  |  |  |
| Inactive | 6.7 (45) | 5.1 (147) | 10.3 (33) | 6.8 (77) | 11.3 (12) | 13.5 (46) |
| Moderately inactive | 23.2 (157) | 22.9 (653) | 31.8 (102) | 28.7 (325) | 31.1 (33) | 29.9 (102) |
| Moderately active | 26.5 (179) | 28.2 (804) | 25.5 (82) | 24.4 (276) | 21.7 (23) | 27.6 (94) |
| Active | 43.6 (295) | 43.8 (1,252) | 32.4 (104) | 40.1 (453) | 35.8 (38) | 29.0 (99) |
| **Smoking behavior, % (n)** |  |  |  |  |  |  |
| Current | 20.7 (140) | 21.4 (612) | 29.9 (96) | 28.8 (326) | 41.5 (44) | 38.4 (131) |
| Former | 39.5 (267) | 35.4 (1,010) | 33.0 (106) | 28.6 (323) | 25.5 (27) | 23.2 (79) |
| Never | 39.8 (269) | 43.2 (1,234) | 37.1 (119) | 482 42.6 | 34 32.1 | 131 38.4 |
| **Alcohol consumption, g/day, mean (sd)** | 10.5 (13.5) | 9.2 (11.7) | 9.9 (14.8) | 9.2 (13.9) | 10.2 (13.0) | 8.1 (12.5) |
| **Diabetes, % (n)** | 0.3 (2) | 0.2 (7) | 3.7 (12) | 2.6 (29) | 12.3 (13) | 14.7 (50) |
| **Systolic blood pressure, mmHg, mean (sd)** | 120.9 (13.7) | 119.8 (13.7) | 142.4 (18.2) | 141.5 (17.7) | 158.0 (18.8) | 158.8 (18.5) |
| **Total cholesterol, mmol/L, mean (sd)** | 5.6 (1.0) | 5.7 (1.0) | 6.3 (1.0) | 6.4 (1.0) | 6.8 (0.9) | 6.7 (1.2) |
| **HDL cholesterol, mmol/L, mean (sd)** | 1.6 (0.4) | 1.6 (0.4) | 1.4 (0.4) | 1.4 (0.4) | 1.2 (0.3) | 1.2 (0.3) |
| **Body mass index, kg/m², mean (sd)** | 25.2 (3.7) | 25.1 (3.8) | 27.3 (4.2) | 27.2 (4.2) | 28.5 (3.8) | 28.1 (4.5) |
| **Framingham risk score, median (IQR)‡** | 8 (6-11) | 8 (5-10) | 15 (14-16) | 15 (14-16) | 19 (18-21) | 19 (18-21) |
| **The following data were collected at time of breast cancer diagnosis or reference (t1)** | | | | |  |  |
| **Age at t1, yr, median (IQR)** | 60 (53-65) | 60 (54-65) | 66 (61-71) | 66 (62-71) | 69 (64-73) | 69 (64-73) |
| **Year of t1, n (%)** |  |  |  |  |  |  |
| 1993 - 1999 | 25.1 (170) | 25.8 (737) | 29.0 (93) | 27.1 (306) | 30.2 (32) | 32.0 (109) |
| 2000 - 2005 | 35.5 (240) | 37.2 (1,062) | 42.0 (135) | 39.4 (446) | 43.4 (46) | 44.0 (150) |
| 2006 - 2010 | 39.4 (266) | 37.0 (1,057) | 29.0 (93) | 33.5 (379) | 26.4 (28) | 24.0 (82) |
| **Time between t0 and t1, yr, median (IQR)** | 8 (4-12) | 8 (4-12) | 7 (3-11) | 8 (4-11) | 6 (3-10) | 6 (3-10) |
| **Follow-up time since t1 (until end of study), yr, median (IQR)** | 5 (2-9) | 6 (2-10) | 6 (3-9) | 6 (3-10) | 5 (2-8) | 7 (3-10) |
| **History of cardiovascular disease at t1, n (%)** | 3.8 (26) | 3.1 (89) | 9.3 (30) | 90 8.0 | 11.3 (12) | 11.7 (40) |
| Abbreviations: IQR = Interquartile Range, sd = standard deviation, yr = years | | | |  |  |  |
| * Women were matched by 1. age at EPIC-NL enrollment (age at t0) and 2. time between EPIC-NL enrollment and breast cancer diagnosis (t1-t0) | | | | | | |
| † Low educational level: lower vocational training or primary school | | | | | | |
| ‡ Framingham risk score is based on age at original cohort enrolment (age at t0), smoking behavior, diabetes, systolic blood pressure, and total and HDL cholesterol (all measured at t0) | | | | | | |

**Table B.** The risk of cardiovascular disease hospitalization and/or death following breast cancer for the total study population and by low, intermediate, or high Framingham risk until December 31, 2010.
*Sensitivity analysis: women with a history of cardiovascular disease at t1 were excluded.*

|  | **Number of women** | **Total PY** | **CVD (%)*** | **CVD per 100 PY** | **Unadjusted HRs†** | **Adjusted HRs†‡** | **Adjusted HRs†§** |
| --- | --- | --- | --- | --- | --- | --- | --- |
| **CVD event (hospitalization or death)** | |  |  |  |  |  |  |
| **Total study population (n = 5,144)** | |  |  |  |  |  |  |
| Women without breast cancer | 4,109 | 28,035 | 320 (7.8) | 1.2 | 1 | 1 | 1 |
| Women with breast cancer | 1,035 | 6,401 | 89 (8.6) | 1.5 | 1.22 (0.97-1.54) | 1.16 (0.92-1.47) | 1.16 (0.92-1.47) |
| **Framingham risk < 10% (n = 3,417)** | |  |  |  |  |  |  |
| Women without breast cancer | 2,767 | 18,518 | 129 (4.7) | 0.7 | 1 | 1 | 1 |
| Women with breast cancer | 650 | 3783 | 39 (6.0) | 1.1 | 1.48 (1.03-2.12) | 1.47 (1.03-2.11) | 1.46 (1.02-2.10) |
| **Framingham risk 10% - 20% (n = 1,332)** | | | | | | | |
| Women without breast cancer | 1,041 | 7,187 | 123 (11.7) | 1.8 | 1 | 1 | 1 |
| Women with breast cancer | 291 | 2031 | 27 (9.3) | 1.4 | 0.80 (0.52-1.22) | 0.82 (0.53-1.25) | 0.82 (0.54-1.25) |
| **Framingham risk > 20% (n = 395)** | |  |  |  |  |  |  |
| Women without breast cancer | 301 | 2287 | 65 (21.5) | 3.1 | 1 | 1 | 1 |
| Women with breast cancer | 94 | 584 | 23 (24.5) | 4.3 | 1.33 (0.82-2.16) | 1.34 (0.82-2.17) | 1.33 (0.82-2.17) |
| **Death from CVD** |  |  |  |  |  |  |  |
| **Total study population (n = 5,144)** | |  |  |  |  |  |  |
| Women without breast cancer | 4,109 | 29,207 | 51 (1.2) | 0.2 | 1 | 1 | - |
| Women with breast cancer | 1,035 | 6,717 | 21 (2.0) | 0.3 | 1.83 (1.10-3.05) | 1.73 (1.04-2.87) |  |
| Abbreviations: CVD = cardiovascular disease, HRs = hazard ratios, PY = person-years  * Row percentages of number of women  † Cox proportional hazard models Models on the total study population are adjusted for Framingham risk score‡ (and body mass index§).  Models stratified by Framingham risk category are adjusted for age at breast cancer diagnosis or reference (age at t1)‡ and body mass index§. | | | | | | | |

**Table C.** The risk of cardiovascular disease hospitalization and/or death following breast cancer in the total study population and by low, intermediate, and high Framingham risk until at most December 31, 2010

*Competing risk analyses: breast cancer as competing risk*

|  | **Number of women** | **Total PY** | **CVD (%)*** | **CVD per 100 PY** | **Unadjusted HRs†** | **Adjusted HRs†‡** | **Adjusted HRs†§** |
| --- | --- | --- | --- | --- | --- | --- | --- |
| **CVD event (hospitalization or death)** | | | | | | | |
| **Total study population (n = 5,431)** | | | | | | |  |
| Women without breast cancer | 4,328 | 28,035 | 325 (7.5) | 1.2 | 1 | 1 | 1 |
| Women with breast cancer | 1,103 | 6,401 | 92 (8.3) | 1.4 | 1.14 (0.91-1.44) | 1.08 (0.86-1.37) | 1.08 (0.86-1.36 |
| **Framingham risk < 10% (n = 3,532)** | |  |  |  |  |  |  |
| Women without breast cancer | 2,856 | 18,518 | 129 (4.5) | 0.7 | 1 | 1 | 1 |
| Women with breast cancer | 676 | 3783 | 39 (5.8) | 1 | 1.352 (0.95-1.93) | 1.36 (0.95-1.94) | 1.34 (0.94-1.93) |
| **Framingham risk 10% - 20% (n = 1,452)** | | | | | | | |
| Women without breast cancer | 1,131 | 7,187 | 124 (10.9) | 1.7 | 1 | 1 | 1 |
| Women with breast cancer | 321 | 2031 | 30 (9.3) | 1.5 | 0.80 (0.54-1.20) | 0.81 (0.54-1.22) | 0.81 (0.54-1.22) |
| **Framingham risk > 20% (n = 447)** | | | | | | | |
| Women without breast cancer | 341 | 2287 | 68 (19.9) | 3 | 1 | 1 | 1 |
| Women with breast cancer | 106 | 584 | 23 (21.7) | 3.9 | 1.17 (0.73-1.89) | 1.16 (0.72-1.88) | 1.16 (0.72-1.87) |
| **Death from CVD** |  |  |  |  |  |  |  |
| **Total study population (n = 5,431)** | | | | | | | |
| Women without breast cancer | 4,328 | 29,207 | 57 (1.3) | 0.2 | 1 | 1 | - |
| Women with breast cancer | 1,103 | 6,717 | 24 (2.2) | 0.4 | 1.72 (1.07-2.77) | 1.58 (0.98-2.55 |  |
| Abbreviations: CVD = cardiovascular disease, HRs = hazard ratios, PY = person-years  * Row percentages of number of women  † Models by the Fine and Gray method  Models on the total study population are adjusted for Framingham risk score‡ (and body mass index§).  Models stratified by Framingham risk category are adjusted for age at breast cancer diagnosis or reference (age at t1)‡ and body mass index§. | | | | | | | |
